# Network Pharmacology and Molecular Docking Analysis Reveal Insights into the Molecular Mechanism of Shengma-Gegen Decoction on Monkeypox

**DOI:** 10.3390/pathogens11111342

**Published:** 2022-11-13

**Authors:** Liujiang Dai, Guizhong Zhang, Xiaochun Wan

**Affiliations:** 1 Guangdong Immune Cell Therapy Engineering and Technology Research Center, Center for Protein and Cell-Based Drugs, Institute of Biomedicine and Biotechnology, Shenzhen Institute of Advanced Technology, Chinese Academy of Sciences, Shenzhen 518055, China; 2 University of Chinese Academy of Sciences, Beijing 100864, China

**Keywords:** monkeypox, network pharmacology, molecular docking

## Abstract

Background: A new viral outbreak caused by monkeypox has appeared after COVID-19. As of yet, no specific drug has been found for its treatment. Shengma-Gegen decoction (SMGGD), a pathogen-eliminating and detoxifying agent composed of four kinds of Chinese herbs, has been demonstrated to be effective against several viruses in China, suggesting that it may be effective in treating monkeypox, however, the precise role and mechanisms are still unknown. Methods: Network pharmacology was used to investigate the monkeypox-specific SMGGD targets. These targets were analyzed via String for protein-to-protein interaction (PPI), followed by identification of hub genes with Cytoscape software. Function enrichment analysis of the hub targets was performed. The interactions between hub targets and corresponding ligands were validated via molecular docking. Results: Through screening and analysis, a total of 94 active components and 8 hub targets were identified in the TCM-bioactive compound-hub gene network. Molecular docking results showed that the active components of SMGGD have strong binding affinity for their corresponding targets. According to functional analysis, these hub genes are mainly involved in the TNF, AGE-RAGE, IL-17, and MAPK pathways, which are linked to the host inflammatory response to infection and viral replication. Therefore, SMGGD might suppress the replication of monkeypox virus through the MAPK signaling pathway while also reducing inflammatory damage caused by viral infection. Conclusion: SMGGD may have positive therapeutic effects on monkeypox by reducing inflammatory damage and limiting virus replication.

## 1. Introduction

Monkeypox virus, first isolated from a monkey in 1958, belongs to the Orthopoxvirus, a kind of double-stranded DNA virus [1]. An unexpected outbreak of monkeypox has emerged, posing a threat to people all around the world [2,3,4,5]. It is likely to become the next threat after novel coronavirus pneumonia (COVID-19) [3,6]. As of 4 October 2022, a total of 68,998 laboratory confirmed and 3203 probable monkeypox virus cases, including 26 deaths, have been reported by 107 countries or regions [7]. The virus can be transmitted through sexual contact, sore contact, scab contact, body fluid contact, and bedding/clothing contact [8]. People infected by monkeypox have symptoms like rash, fever, chills, lymphadenopathy, headache, muscle aches and backache, or others [9]. Historical data have shown that smallpox vaccination with vaccinia virus was approximately 85% effective against monkeypox [10]. As smallpox has been eradicated since 1978 due to widespread vaccination programmes, the vaccination programmes were abolished thereafter. Now, most people worldwide are not immune to smallpox, let alone monkeypox. Tecovirimat is an antiviral drug which used to be the only FDA approved drug for the treatment of smallpox. Recently, it was approved for monkeypox treatment. However, this drug was approved without enough clinical trials [11,12]. In the future, clinical trials should be carried out to prove its safety and efficacy on humans. Currently, it is urgent to find other effective approaches for monkeypox treatment.

Shengma-Gegen decoction (SMGGD), which is composed of four kinds of Chinese herbal medicines, such as Shengma (*Cimicifugae Rhizoma*), Gegen (*Radix Puerariae*), Baishao (*Paeoniae Radix Alba*), and Gancao (*Glycyrrhiza uralensis*, licorice), was originally used against measles in children for many hundreds of years in China. SMGGD has been proved against different viruses including measles virus [13], human respiratory syncytial virus (HRSV) [14], and hepatitis B virus (HBV) [15]. With the increasing spread of monkeypox worldwide, In June 2022, the Diagnosis and Treatment Guideline of Monkeypox was released by the National Health Commission and National Administration of Traditional Chinese Medicine of the People’s Republic of China, in which SMGGD and other traditional Chinese medicine (TCM) formulas were recommended to treat monkeypox according to different symptoms [16]. It notes patients can take SMGGD when they get a fever due to monkeypox infection.

Network pharmacology was proposed by Andrew Hopkins in 2007 [17]. It acts as an excellent analysis platform by integrating data from different sources. Active compounds from given Chinese herbs are screened, followed by identification of their target genes. After the disease-related genes and targets of active compounds are intersected, the TCM-compound-target gene-disease network will be constructed [18]. It helps us to understand the effects as well as the potential mechanisms of TCM on disease. Molecular docking is an established method based on a computer simulation structure. It can be achieved by using AutoDock and PyMOL to simulate docking between ligands and receptors. The results of molecular docking can be used to verify the binding ability between active compounds and key targets and improve the accuracy of the target network [19].

In this study, network pharmacology and molecular docking strategies were used to explore the mechanism of SMGGD on monkeypox infection. According to the preliminary analysis results, SMGGD reduces inflammatory damage caused by AGE-RAGE, TNF, and IL-17 signaling and restricts virus replication via the MAPK pathway.

## 2. Materials and Methods

### 2.1. Screen of SMGGD Bioactive Compounds

**Figure 1:**
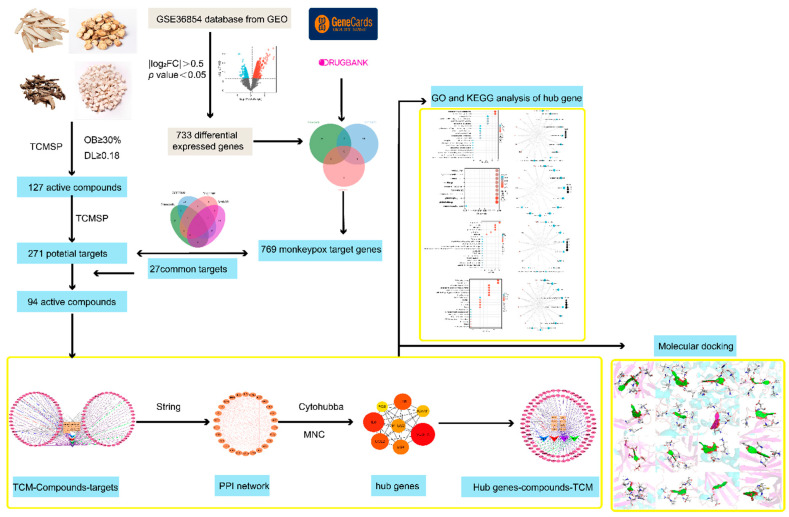
The workflow of the study based on a network pharmacology approach.

By using “Shengma”, “Gegen”, “Baisao”, “Gancao” as keywords, the bioactive compounds from the TCMSP platform (https://old.tcmsp-e.com/tcmsp.php, accessed on 30 July 2022) were screened under the following standard criteria: OB ≥ 30% and DL ≥ 0.18 [20]. However, some main compounds of SMGGD were not included under the criteria. To accurately study how bioactive compounds of SMGGD treat monkeypox, the main compounds of SMGGD were included (Supplementary Table S1). The flow chart of this study is shown in Figure 1.

### 2.2. Target Genes Related to Bioactive Compounds

The target genes were collected according to the screened active components of SMGGD from the TCMSP platform (https://old.tcmsp-e.com/tcmsp.php, accessed on 6 August 2022). Then, the target genes were converted into corresponding gene symbols of the “Homo sapiens” species using the UniProt database (https://www.uniprot.org/, accessed on 7 August 2022) [21].

### 2.3. The Specific Genes of Monkeypox

GSE36854 was downloaded from the GEO database (https://www.ncbi.nlm.nih.gov/geo/, accessed on 28 September 2022). The differentially expressed genes (DEGs) were obtained via GEO2R online tool analysis [R (3.2.3), Biobase (2.30.0), GEOquery (2.40.0), limma (3.26.8), https://www.ncbi.nlm.nih.gov/geo/geo2r/, accessed on 28 September 2022]. Monkeypox-related targets were obtained under the following criteria: *p* value < 0.05, |log_2_FC| > 0.5. The Volcano Plot was produced by Xiantao Xueshu web tool (https://www.xiantao.love, accessed on 5 October 2022) in which R software (3.6.3) and ggplot2 (3.3.3) package were employed [22]. Another set of monkeypox genes were download from Genecards Database (https://www.genecards.org, accessed on 10 September 2022) using the keywords monkeypox, monkeypox virus [23]. Additionally, DrugBank (https://www.drugbank.com, accessed on 10 September 2022) was used to obtain more related genes [24].

### 2.4. Monkeypox Specific Genes Related to Active Compounds of SMGGD

By comparing the monkeypox-associated genes with targets of the active compounds of SMGGD, the monkeypox-specific SMGGD target genes were identified.

### 2.5. Network Construction

#### 2.5.1. The Construction of the Pharmacological Network

A file containing a network of the above common targets and corresponding active compounds was created. An annotation file containing the TCM and the compounds was created as well. Then, the files were imported into Cytoscape software (version 3.9.1), and a TCM-compound-target network was constructed.

#### 2.5.2. Construction of Protein–Protein Interaction (PPI) Network and Screening of Core Targets

A total of 27 monkeypox-specific SMGGD target genes were imported into the String database (https://string-db.org/, accessed on 4 October 2022). The species was set as “Homo sapiens”, all PPI relationships were obtained, and the confidence score was ≥0.4 [25]. Then, the obtained data file was imported into Cytoscape software to generate the PPI network diagram of monkeypox-specific genes regulated using active components of SMGGD. The Cytohubba plug-in was used to identify the hub gene, and the top 8 genes generated via the maximum neighborhood component (MNC) method were regarded as the hub genes [26]. For each node in an interactive network, the degree represents the number of edges between one node and other nodes in the network, which measures the number of connections to other nodes and reflects the importance of the node [27].

#### 2.5.3. Construction of TCM-Bioactive Compound-Hub Genes Network

According to the active components related to the hub genes, a TCM-bioactive compound-hub gene network was constructed.

### 2.6. Gene Ontology (GO) Functional Annotation and Kyoto Encyclopedia of Genes and Genomes (KEGG) Pathway Analysis

To systematically understand the mechanisms of SMGGD in the treatment of monkeypox, GO and KEGG analysis of hub targets was performed. The top 20 terms and terms with a *p* value of less than 0.05 were visualized in this study. The above analysis was completed using the Xiantao Xueshu web tool (https://www.xiantao.love, accessed on 5 October 2022) in which R software (3.6.3) and R-language packages, including “ggplot2 (3.3.3)” and “ClusterProfiler (3.14.3)”, were applied [22].

### 2.7. Molecular Docking between Compounds and Targets

Semi-flexible docking was carried out using AutoDock Tools (ADT) software (version 1.5.6) to verify the binding ability of bioactive components to key targets [28,29]. The specific operations were as follows:

1. Preparation of receptors: The 3D structure of 8 hub targets (receptors) were obtained from the RCSBPDB database (https://www.rcsb.org/, accessed on 4 October 2022) and saved in PDB format. The obtained 3D structures (PDB format) were further processed by removing water molecules (command of “remove solvent”) and the ligand (command of “remove organic”) using PyMOL sofware (version 2.2.0).
2. Preparation of ligands: 2D structure files (SDF format) of active compounds were downloaded from the PubChem website (https://pubchem.ncbi.nlm.nih.gov/, accessed on 4 October 2022) [30]. Then, the files were converted into PDB format using Open Babel software (version 2.4.1).
3. Molecular docking: Hydrogen and Gasteiger charges were added to the above receptors and ligands using ADT software before they were saved in PDBQT format. The AutoGrid Tool of the ADT software was used to set the docking frame parameters including setting the grid box containing the entire receptor. The parameter was set as the Lamarckian genetic algorithm (LGA) to generate 10 docking results for each ligand with the corresponding receptor. All of the docking results were visualized via PyMOL software.

## 3. Results

### 3.1. SMGGD Bioactive Compounds and Related Targets Selection

A total of 19 compounds from Shengma, 7 compounds from Gegen, 15 compounds from Baisao, and 94 compounds from Gancao were found via the TCMSP platform. A total of 127 active components were selected for further analysis after removal of the duplicates. The corresponding targets of the SMGGD were obtained via the TCMSP database as well. The corresponding targets of Shengma, Gengen, Baisao, and Gancao filtered out by TCMSP were 47, 134, 77, and 218 respectively. After removing the repeated targets, a total of 271 potential targets were obtained.

### 3.2. Compound Target Network and Compound Target Disease Network

**Figure 2:**
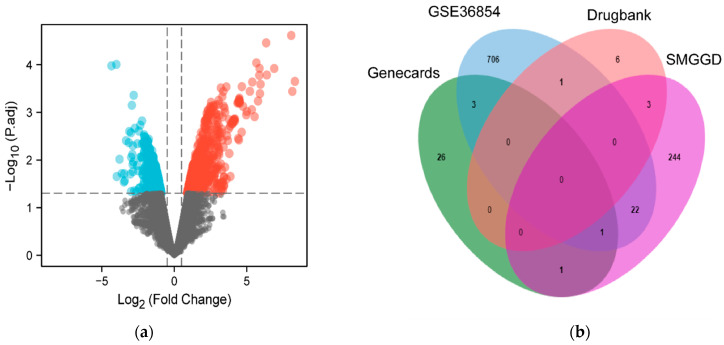
Collection of monkeypox associated genes. (**a**) The gene volcano map shows the gene distribution from GSE36854. Red and light blue represent up-regulated genes and down-regulated genes (*p* value < 0.05, |log_2_FC| > 0.5), the black indicates no significant difference. (**b**) The Venn diagram shows the genes from three databases (GSE36854, GeneCards, and DrugBank) and targets from SMGGD. The 27 common targets were chosen for further analysis.

In order to obtain monkeypox-related genes, GSE36854 were downloaded and analyzed from the GEO database. In that study, the differentially expressed genes (DEGs) were obtained by performing microarray analysis on the human HeLa cells infected with monkeypox virus and uninfected cells. In this study, the DEGs were obtained via GEO2R online tool analysis. According to the criteria, which was *p* value < 0.05, |log_2_FC| > 0.5, a total of 418 upregulated genes and 315 downregulated genes were identified (Figure 2a). More monkeypox-related genes were obtained from the Genecards and Drugbank databases; 31 and 10 genes were found individually.

After merging and deleting the duplicate values from all the above sources, a total of 769 monkeypox related genes were obtained (Figure 2b). By comparing the 769 monkeypox-associated genes with 271 targets of the SMGGD, 27 common targets were identified (Figure 2b).

**Figure 3:**
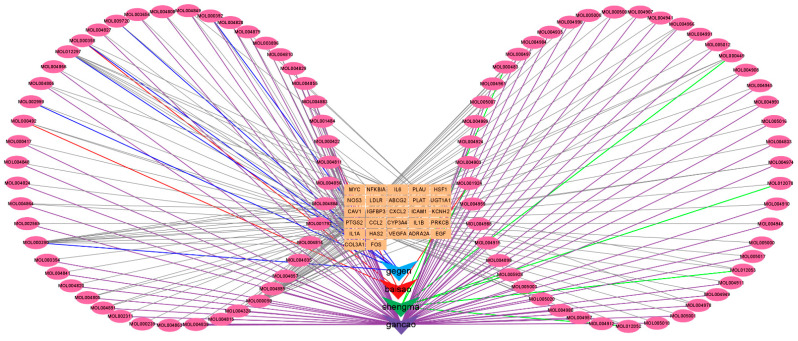
TCM-bioactive compounds-common targets network.

Next, the TCM-compounds-common targets network was constructed using Cytoscape software. The network consists of 125 nodes (including 27 target nodes, 94 compound nodes, and 4 TCM nodes) and 254 edges, in which the circle node represents the active compound, the V-shaped node represents TCM, and the rectangle node represents the target (Figure 3 and Supplementary Table S2). 7 key active components with degrees ≥ 4 were identified as quercetin (MOL000098), daidzein (MOL000390), puerarin (MOL012297), kaempferol (MOL000422), beta-sitosterol (MOL000358), stigmasterol (MOL000449), and naringenin (MOL004328).

**Figure 4:**
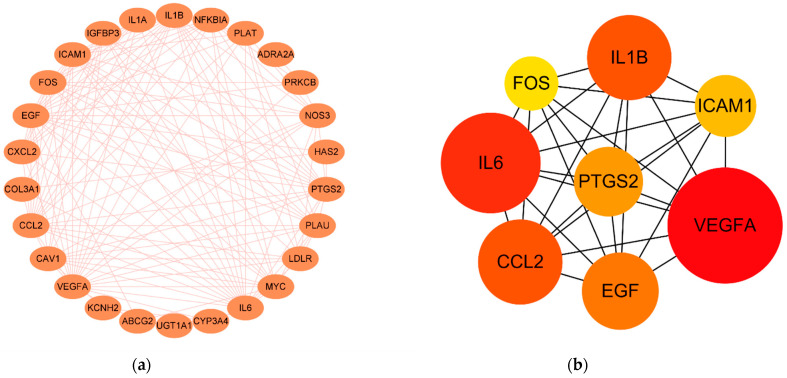

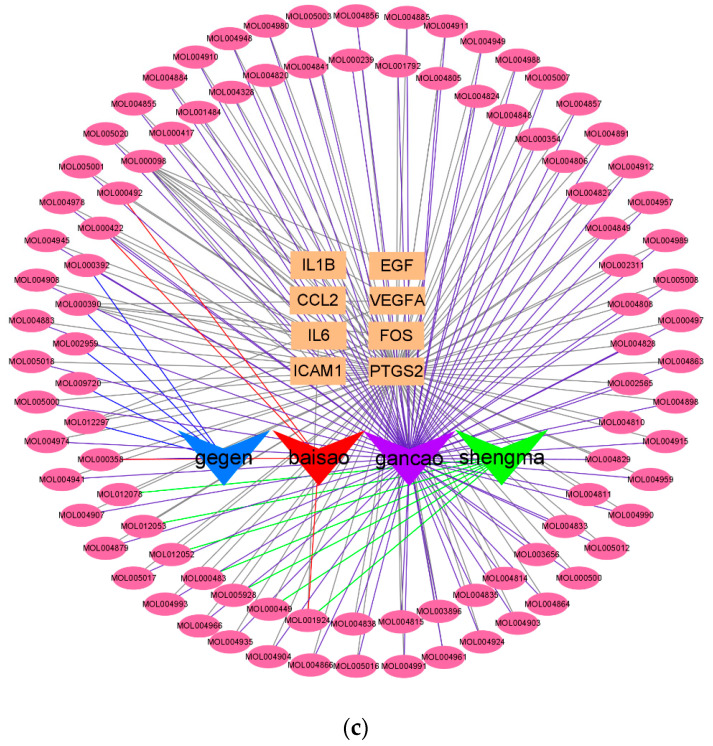
TCM-bioactive compound-hub gene network. (**a**) PPI networks of monkeypox-specific SMGGD targets. (**b**) 8 hub genes identified via MNC method. (**c**) TCM-bioactive compound-hub gene network.

The 27 monkeypox-specific SMGGD target genes were introduced into the String database online service platform to construct a PPI network consisting of 26 nodes and 141 interaction edges (Figure 4a). All the obtained PPI relationships were imported into Cytoscape software. Then, the Cytohubba plug-in was used to screen the top 8 hub genes. VEGFA, IL1B, EGF, CCL2, FOS, PTGS2, IL6, and ICAM1 were found using the MNC method (Figure 4b). Most of the hub genes were found upregulated upon monkeypox treatment in the GSE36854 data except for downregulated EGF.

PTGS2 was the highest node and had the degree value of 93. IL6, VEGFA, FOS, and ICAM1 had a value of 3, respectively. The rest of the hub genes only had one degree value. Next, the TCM-bioactive compound-hub gene network, which had 106 nodes (including 8 target nodes, 94 compound nodes, and 4 TCM nodes) and 206 edges, was constructed (Figure 4c). The details of the 8 hub genes are listed in Supplementary Table S3.

### 3.3. GO and KEGG Enrichment Analysis

**Figure 5:**
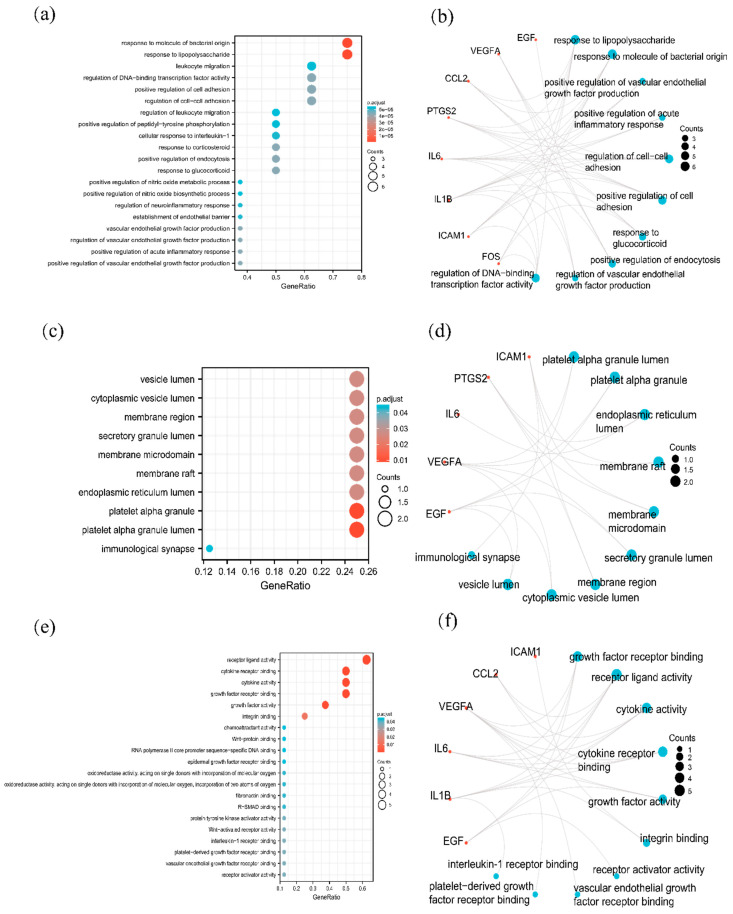

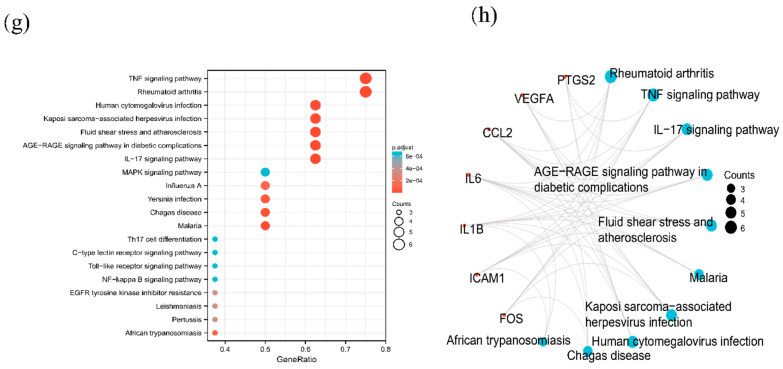
Bubble maps of GO-BP, GO-CC, GO-MF, and KEGG enrichment analysis and related gene circle maps. (**a**) The top 20 terms of GO-BP. (**b**) A circle diagram showing the top 10 BP terms and related genes. (**c**) The top 10 terms of GO-CC. (**d**) A circle diagram showing the top 10 CC terms and related genes. (**e**) The top 20 terms of GO-MF. (**f**) A circle diagram showing the top 10 MF terms and related genes. (**g**) The top 20 enriched pathways after KEGG analysis. (**h**) A circle diagram showing the top 10 enriched pathways and related genes.

GO analysis of the above 8 hub genes was performed. The top BP, CC, and MF terms are shown in bubble charts (Figure 5a,c,e). The top 10 enrichment results with their targets are shown in Figure 5b,d,f. More detailed information is shown in Supplementary Table S4.

The top 5 enrichment analysis results of BP terms were as follows: response to lipopolysaccharide (GO:0032496), response to molecule of bacterial origin (GO:0002237), positive regulation of vascular endothelial growth factor production (GO:0010575), positive regulation of acute inflammatory response (GO:0002675), regulation of cell-cell adhesion (GO:0022407). The top 5 CC terms were as follows: platelet alpha granule lumen (GO:0031093), platelet alpha granule (GO:0031091), endoplasmic reticulum lumen (GO:0005788), membrane raft (GO:0045121), membrane microdomain (GO:0098857). The top 5 MF terms were as follows: growth factor receptor binding (GO:0070851), receptor ligand activity (GO:0048018), cytokine activity (GO:0005125), cytokine receptor binding (GO:0005126), growth factor activity (GO:0008083).

To clarify the role of the hub genes in signal transduction, KEGG enrichment analysis was carried out. The top KEGG analysis is shown in Figure 5g,h. The details of the pathways and enriched genes were listed in Table 1. The top 5 enriched pathways according to the adjusted *p* value were rheumatoid arthritis (hsa05323), the TNF signaling pathway (hsa04668), the IL-17 signaling pathway (hsa04657), the AGE-RAGE signaling pathway in diabetic complications (hsa04933), fluid shear stress and atherosclerosis (hsa05418).

### 3.4. Molecular Docking

**Figure 6:**
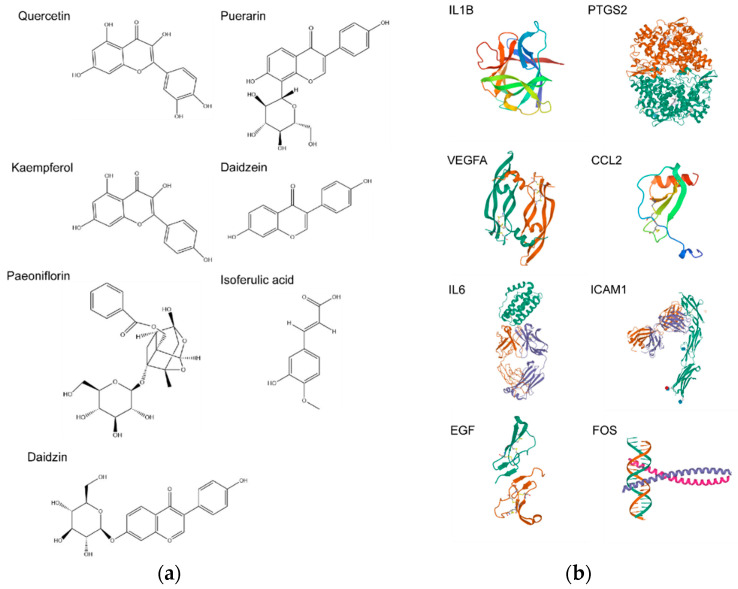
The structure of compounds and their targets. (**a**) Structures of key compounds. (**b**) Three-dimensional structures of 8 hub targets.

Next, the interactions between the key targets and related compounds were validated via molecular docking. Puerarin (MOL012297), daidzein (MOL000390), quercetin (MOL000098), and kaempferol (MOL000422) could target multiple proteins in the TCM-bioactive compound-hub gene network. Especially, quercetin targeted all of the hub genes. Puerarin could target VEGFA, FOS, and PTGS2. Daidzein could target VEGFA, FOS, PTGS2, IL6, and ICAM1. Kaempferol could target PTGS2 and ICAM1. The rest of the compounds targeted PTGS2, except that paeoniflorin (MOL001924) targeted IL6. Puerarin, daidzein, quercetin, and kaempferol therefore were chosen as ligands (Figure 6a), and the corresponding targets were used as receptors (Figure 6b). As paeoniflorin, daidzin (MOL009720) and isoferulic acid (MOL005928) are the main compounds of SMGGD [31,32], the three compounds and their corresponding targets were chosen for molecular docking as well. Then, the molecular docking between ligands and receptors were analyzed via AutoDock Tools software. Generally, the binding ability between a receptor and a ligand based on the value of the binding affinity was evaluated. If the energy is less than −5 kcal/mol, it indicates the receptor has a certain binding ability with the ligand. The lower the binding energy is, the more sable the binding conformation will be [33,34].

**Figure 7:**
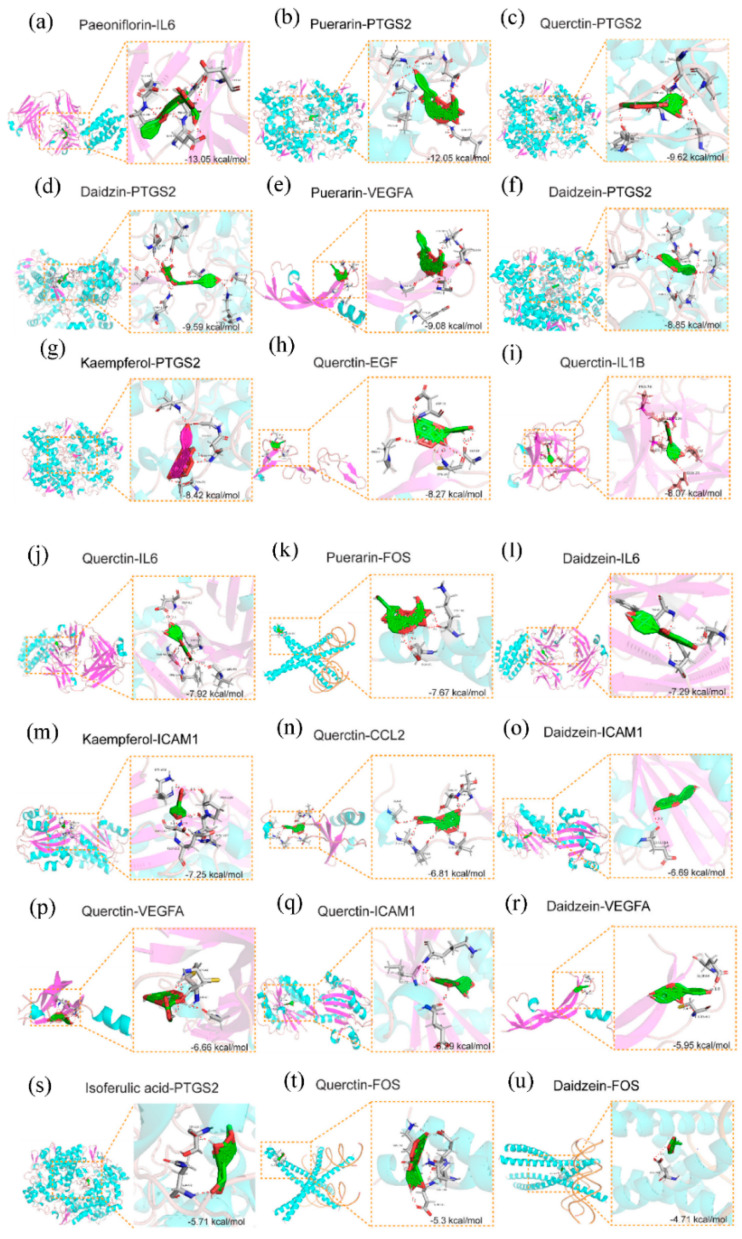
Molecular docking of active ingredients and hub genes.

All the molecular docking results are listed in Table 2. Molecular docking analysis demonstrated that paeoniflorin and IL6 (−13.05 kcal/mol), puerarin and PTGS2 (−12.05 kcal/mol), quercetin and PTGS2 (−9.62 kcal/mol), daidzin and PTGS2 (−9.59 kcal/mol), puerarin and VEGFA (−9.08 kcal/mol), daidzein and PTGS2 (−8.85 kcal/mol), kaempferol and PTGS2 (−8.42 kcal/mol), quercetin and EGF (−8.27 kcal/mol), and quercetin and IL1B (−8.07 kcal/mol) showed stronger binding affinity that other groups. Especially, the docking score of paeoniflorin and IL6 was the lowest among all the groups, which suggests the strongest binding affinity. Therefore, the above listed ingredients may be potential compounds in treating monkeypox-related symptoms. All the molecular docking results were displayed in Figure 7.

## 4. Discussion

**Figure 8:**
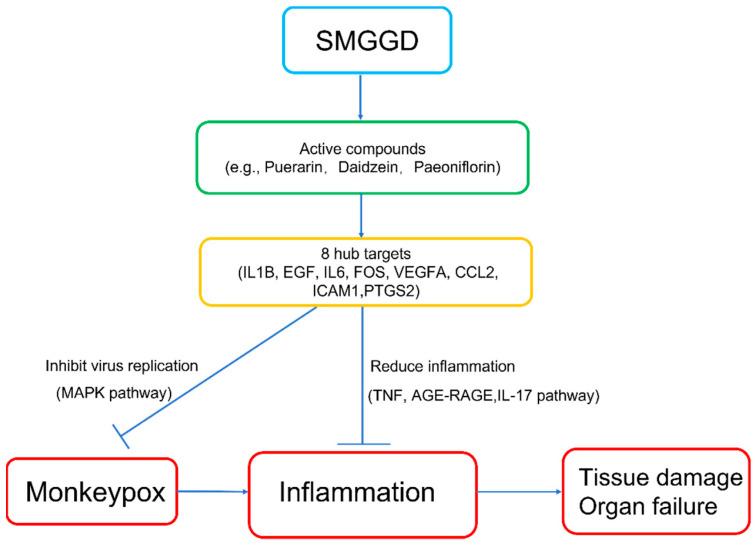
Potential mechanism of SMGGD on monkeypox treatment.

An unexpected outbreak of monkeypox has emerged. There is no specific medication established for its treatment so far. It is urgent to find safe and effective drugs to treat monkeypox. TCM has accumulated abundant clinical experiences and effective formulas on the prevention and treatment of epidemic diseases. It has been proved to cure viral diseases by inhibiting virus replication. Previous clinical experiences of TCM on treating SARS and SARS-CoV-2 hint that TCM intervention might have positive efficacy against monkeypox. Moreover, the Chinese government has released the Diagnosis and Treatment Guideline of Monkeypox in which TCM is suggested to treat this disease. This study aimed to discover the efficacy of SMGGD for treating monkeypox using a network pharmacology approach. After identifying the common targets between monkeypox-related genes and targets of bioactive compounds from SMGGD, 8 hub genes were obtained via a PPI network followed by Cytoscape software analysis. GO and KEGG enrichment analysis showed the hub targets were associated with acute inflammatory response and cytokine activity and virus infection (Figure 5g,h and Table 1). SMGGD might treat monkeypox through anti-inflammatory and antiviral mechanisms (Figure 8). 

A total of 94 active components and 8 hub targets were identified in the TCM-bioactive compound-hub gene network. Among these active components, puerarin, daidzein, and daidzin are the main compounds in *Radix Puerariae* [31], isoferulic acid is the main compound in *Cimicifugae Rhizoma* [32], paeoniflorin not only presents in *Cimicifugae Rhizoma*, it is one of the main compounds in *Paeoniae Radix Alba* [35], quercetin presents in *Glycyrrhiza uralensis*, and kaempferol presents in *Glycyrrhiza uralensis* and *Paeoniae Radix Alba*. Wang et al. discovered puerarin exhibited antiviral activity against influenza virus by inhibiting the NA activity of the virus and blocking the nuclear output of key proteins [36]. Puerarin was also found to have anti-inflammatory effects against viral diseases [37]. He et al. found daidzein inhibited hepatitis C virus replication and enhanced the antiviral effect of IFN-α by downregulating microRNA-122 expression [38]. Daidzein also exerted anti-inflammatory activity by suppressing pro-inflammatory chemokine Cxcl2 transcription [39]. Zhang et al found daidzin displayed antiviral activity by binding with translational frameshift site RNA of Japanese encephalitis virus (JEV) to form non-covalent complexes [40]. Daidzin possessed anti-inflammatory activity as well [41]. Isoferulic acid was reported to exert an anti-inflammatory effect by inhibiting IL8 or macrophage inflammatory protein-2 (MIR-2) production induced by virus infection [42,43]. Yu et al. found paeoniflorin protected against influenza A virus-induced acute lung injury by reducing pro-inflammatory cytokine production and lung collagen deposition [44]. Quercetin is a polyphenolic flavonoid, and other studies showed it indeed has an anti-viral activity against different viruses such as hepatitis C virus, influenza virus, and dengue virus [45,46,47,48,49]. Mechanism studies showed quercetin inhibited influenza infection by interacting with the HA2 subunit [46]. Kaempferol possesses a chemical structure similar to that of quercetin. Other studies showed that quercetin and kaempferol had antiviral activities against SARS-CoV-2 which were mediated through inhibiting protein kinase B, phosphorylating of protein kinase, and blocking of the 3a channel [50,51,52]. The two compounds also regulated immune responses by reducing pro-inflammatory cytokines and enhancing anti-inflammatory cytokines, which have been proved to have a positive effect on COVID-19 treatment [53,54,55,56]. Monkeypox virus replicates at the initial infection site, which causes an inflammatory response [57]. The presence of the virus greatly affects T-cell-mediated cytokine responses [58]. The bioactive compounds from SMGGD might suppress monkeypox by directly inhibiting the virus, as well as reducing inflammation caused by monkeypox infection.

A total of 8 hub targets, including IL1B, EGF, IL6, FOS, VEGFA, CCL2, ICAM1, and PTGS2 were identified in our study. According to the TCM-bioactive compound-hub gene network, it was likely that SMGGD exhibited a therapeutic effect on monkeypox by mainly targeting the 8 proteins. Among the targets, PTGS2, which could be targeted by 93 compounds from SMGGD, ranked the highest. PTGS2 is one of the key enzymes in prostaglandin biosynthesis, related to inflammation and mitosis. One study showed the inhibition of HIV-1, HCV, and HSV by serpin antithrombin III (ATIII) may be the result of downstream synthesis of eicosanoids (including prostaglandin) mediated by PTGS2 [59]. Furthermore, inhibition of PTGS2 activity could alleviate lung infection [60]. Another study indicated the inhibition of PTGS2 could be a potential therapeutic strategy for SARS-CoV-2 infection [61]. Other identified hub targets were associated with immune response and cytokines secretion. For example, FOS is a stress response gene modifying the structure of cells in human development. It can activate an immune response upon bacterial infection [62]. IL6 is one of classic proinflammatory cytokines and directly or indirectly activates a series of different types of cells which further cause secretion of cytokines [63].

The GO term enrichment of hub targets revealed that SMGGD treating monkeypox mainly involved positive regulation of vascular endothelial growth factor production, positive regulation of acute inflammatory response, regulation of cell-cell adhesion, growth factor receptor binding, receptor ligand activity, cytokine activity, cytokine receptor binding, and growth factor activity. The KEGG pathway enrichment analysis of these hub proteins showed they were involved in the TNF signaling pathway, the IL-17 signaling pathway, the AGE-RAGE signaling pathway in diabetic complications, Kaposi sarcoma-associated herpesvirus infection, human cytomegalovirus infection, influenza A, EGFR tyrosine kinase inhibitor resistance, the MAPK signaling pathway, the NF-kappa B signaling pathway, the Toll-like receptor signaling pathway, the C-type lectin receptor signaling pathway, and Th17 cell differentiation (Table 1). The RAGE signaling pathway was reported to have a regulatory effect on diabetes [64]. TNF was reported to regulate viral infection in many studies [65,66]. As a key cytokine in the pathogenesis of inflammation, IL-17 is involved in viral infections as well [67,68,69]. The MAPK pathway is a major cell signaling pathway activated by diverse groups of viruses. It is involved in multiple steps of virus replication [70,71,72]. This is consistent with another study in which the MAPK pathway was found to be enriched in both monkeypox-infected monkey and human models [73]. The GO and KEGG enrichment analysis indicated SMGGD might have positive efficacy against monkeypox by inhibiting inflammation as well as viral replication through these pathways. 

The molecular docking results showed that most of the hub targets and the corresponding ligands had a strong binding affinity, except that the docking scores of daidzein and FOS was more than −5 kcal/mol (−4.71 kcal/mol). All of the docking results are shown in Figure 7. The active compounds from SMGGD had good binding affinity with the corresponding targets (Table 2). Paeoniflorin and IL6 exhibited the strongest binding affinity (−13.05 kcal/mol, Table 2). As mentioned above, paeoniflorin is one of the main compounds in SMGGD. IL6 is a proinflammatory cytokine which also induces secretion of other cytokines. The result illustrated SMGGD might display positive efficacy against monkeypox by inhibiting inflammation. Moreover, PTGS2, which is also associated with inflammation, was found to have good binding affinity with the main compounds of SMGGD including puerarin, daidzin, daidzein, quercetin, and kaempferol. In short, the molecular docking results not only proved that the active compounds of SMGGD had good binding affinity with the hub targets, the results also hinted that SMGGD might have positive efficacy against monkeypox by inhibiting excessive inflammation induced by the disease.

## 5. Conclusions

Taken together, the present study explored the interaction between bioactive compounds of SMGGD and monkeypox-specific SMGGD targets using a network pharmacological approach and bioinformatic analysis, which suggests that SMGGD may have beneficial therapeutic effects on monkeypox by reducing inflammatory damage caused by AGE-RAGE, TNF, and IL-17 signaling and restricting virus replication via the MAPK pathway.

## Figures and Tables

**Table 1:** Top 20 enriched pathways of the hub genes via KEGG analysis.

ID	Description	*p* Value	*p* Adjust	Targets
hsa05323	Rheumatoid arthritis	5.4469 × 10^−11^	6.1006 × 10^−9^	FOS/ICAM1/IL1B/IL6/CCL2/VEGFA
hsa04668	TNF signaling pathway	1.7026 × 10^−10^	9.5347 × 10^−9^	FOS/ICAM1/IL1B/IL6/PTGS2/CCL2
hsa04657	IL-17 signaling pathway	1.0457 × 10^−8^	3.9039 × 10^−7^	FOS/IL1B/IL6/PTGS2/CCL2
hsa04933	AGE-RAGE signaling pathway in diabetic complications	1.4316 × 10^−8^	4.0084 × 10^−7^	ICAM1/IL1B/IL6/CCL2/VEGFA
hsa05418	Fluid shear stress and atherosclerosis	7.5524 × 10^−8^	1.6683 × 10^−6^	FOS/ICAM1/IL1B/CCL2/VEGFA
hsa05144	Malaria	8.9372 × 10^−8^	1.6683 × 10^−6^	ICAM1/IL1B/IL6/CCL2
hsa05167	Kaposi sarcoma-associated herpesvirus infection	3.9113 × 10^−7^	6.2581 × 10^−6^	FOS/ICAM1/IL6/PTGS2/VEGFA
hsa05163	Human cytomegalovirus infection	8.4005 × 10^-7^	1.1761 × 10^−5^	IL1B/IL6/PTGS2/CCL2/VEGFA
hsa05142	Chagas disease	1.6153 × 10^−6^	2.0101 × 10^−5^	FOS/IL1B/IL6/CCL2
hsa05143	African trypanosomiasis	4.8805 × 10^−6^	5.3595 × 10^−5^	ICAM1/IL1B/IL6
hsa05135	Yersinia infection	5.2638 × 10^−6^	5.3595 × 10^−5^	FOS/IL1B/IL6/CCL2
hsa05164	Influenza A	1.2715 × 10^−5^	0.00011867	ICAM1/IL1B/IL6/CCL2
hsa05133	Pertussis	4.3361 × 10^−5^	0.00036078	FOS/IL1B/IL6
hsa05140	Leishmaniasis	4.5098 × 10^−5^	0.00036078	FOS/IL1B/PTGS2
hsa01521	EGFR tyrosine kinase inhibitor resistance	4.8708 × 10^−5^	0.00036368	EGF/IL6/VEGFA
hsa04010	MAPK signaling pathway	0.00010729	0.0006535	EGF/FOS/IL1B/VEGFA
hsa04064	NF-kappa B signaling pathway	0.00011086	0.0006535	ICAM1/IL1B/PTGS2
hsa04620	Toll-like receptor signaling pathway	0.00011086	0.0006535	FOS/IL1B/IL6
hsa04625	C-type lectin receptor signaling pathway	0.00011086	0.0006535	IL1B/IL6/PTGS2
hsa04659	Th17 cell differentiation	0.00012066	0.00067572	FOS/IL1B/IL6

**Table 2:** Binding energy and hydrogen bonds of active ingredients and their target proteins.

Active Ingredients	Hub Targets	Pdb ID	Binding Energy (kcal/mol)	Hydrogen Bonds
paeoniflorin	IL6	4o9h	−13.05	GLU46(A), ASP62(A), GLU99(A), PHE100(A)
puerarin	PTGS2	5ikr	−12.05	VAL228(A), GLN374(A), ASN375(A), GLY533(A), ASN537(A), VAL538(A)
quercetin	PTGS2	5ikr	−9.62	TYR373(A), GLY225(A), VAL228(A), GLY533(A), ASN537(A)
daidzin	PTGS2	5ikr	−9.59	ASN34(A), SER49(A), ALA132(A), GLY135(A), TRP323(A), GLY327(A), LYS459(A)
puerarin	VEGFA	1mjv	−9.08	TYR25(A), GLY59(A), CYS61(A), GLU64(A), LYS107(A)
daidzein	PTGS2	5ikrj	−8.85	VAL228(A), GLN374(A), ASN375(A), GLY533(A), ASN537(A), VAL538(A)
kaempferol	PTGS2	5ikr	−8.42	VAL228(A), TYR373(A), ASN375(A), GLY533(A)
quercetin	EGF	1jl9	−8.27	PRO7(A), ASP11(A), CYS14(A), GLY18(A)
quercetin	IL1B	1hib	−8.07	GLU25(A), PRO78(A), LEU80(A), VAL132(A), LEU134(A)
quercetin	IL6	4o9h	−7.92	LEU45(A), TRP47(A), ASP62(A), THR98(A), PHE100(A)
puerarin	FOS	1fos	−7.67	LYS188(A), GLU191(A)
daidzein	IL6	4o9h	−7.29	LEU45(A), THP47(A), PHE100(A)
kaempferol	ICAM1	2oz4	−7.25	ASP131(A), GLU222(A), ARG227(A), PRO228(A), LYS232(A)
quercetin	CCL2	1dol	−6.81	ALA4(A), ALA7(A), PRO8(A), THR10(A), THR32(A), SER33(A)
daidzein	ICAM1	2oz4	−6.69	GLU284(A)
quercetin	VEGFA	1mjv	−6.66	GLU42(A), TYR45(A), PHE47(A), SER50(A)
quercetin	ICAM1	2oz4	−6.29	ILE-258(A), GLU-284(A), LYS-287(A)
daidzein	VEGFA	1mjv	−5.95	CYS61(A), GLU64(A)
isoferulic acid	PTGS2	5ikr	−5.71	SER126(A), GLN372(A)
quercetin	FOS	1fos	−5.3	LYS188(A), GLU189(A), GLU191(A), LYS192(A)
daidzein	FOS	1fos	−4.71	GLU284

## Data Availability

Not applicable.

## Supplementary Materials

The following supporting information can be downloaded at: https://www.mdpi.com/article/10.3390/pathogens11111342/s1, Table S1, The main bioactive compounds of SMGGD. Table S2, Information for 94 bioactive compounds of SMGGD. Table S3, Top 8 hub genes. Table S4, Details of top GO terms (BP/CC/MF).
