# Supplementary material for: Network Pharmacology and Molecular Docking Analysis Reveal Insights into the Molecular Mechanism of Shengma-Gegen Decoction on Monkeypox
Source: Pathogens. 2022 Nov 13;11(11):1342. doi: 10.3390/pathogens11111342 (PMC9692928; doi:10.3390/pathogens11111342)
Supplement: Supplementary file 1 [file pathogens-11-01342-s001.zip › supplymentary data.pdf]

Supplementary Table S1: The main bioactive compounds of SMGGD

Mol ID	Molecule name	OB%	DL	Source
MOL012297	puerarin	24.03	0.69	gegen
MOL000390	daidzein	19.44	0.19	gegen
MOL009720	daidzin	14.32	0.73	gegen
MOL001924	paeoniflorin	53.87	0.79	baisao shengma
MOL001927	albiflorin	12.09	0.77	baishao
MOL005928	isoferulic acid	50.83	0.06	shengma
MOL011734	Cimifugin	13.49	0.29	shengma
MOL004876	glycyrrhizic acid	19.62	0.11	gancao shengma
MOL004804	glycyrrhetinic acid	22.05	0.74	gancao

Supplementary Table S2: Information for 94 bioactive compounds of SMGGD

Mol ID	Molecule name	Target	OB%	DL	Source
MOL001924	paeoniflorin	IL6	53.87	0.79	baisao shengma
MOL000358	beta-sitosterol	PTGS2 KCNH2	36.91	0.75	baisao gegen
MOL000422	kaempferol	PTGS2 CYP3A4 ICAM1 HAS2	41.88	0.24	baisao gancao
MOL000492	(+)-catechin	PTGS2 HAS2	54.83	0.24	baisao
MOL001484	Inermine	PTGS2	75.18	0.54	gancao
MOL001792	DFV	PTGS2	32.76	0.18	gancao
MOL002311	Glycyrol	PTGS2	90.78	0.67	gancao
MOL000239	Jaranol	PTGS2	50.83	0.29	gancao
MOL002565	Medicarpin	PTGS2	49.22	0.34	gancao
MOL000354	isorhamnetin	PTGS2	49.6	0.31	gancao
MOL003656	Lupiwighteone	PTGS2	51.64	0.37	gancao
MOL003896	7-Methoxy-2-methyl isoflavone	PTGS2	42.56	0.2	gancao
MOL000392	formononetin	PTGS2	69.67	0.21	gancao gegen
MOL000417	Calycosin	PTGS2	47.75	0.24	gancao
MOL004328	naringenin	PTGS2 LDLR UGT1A1	59.29	0.21	gancao
MOL004805	(2S)-2-[4-hydroxy-3-(3-methylbut-2-enyl)phenyl]-8,8-dimethyl-2,3-dihydropyrano[2,3-f]chromen-4-one	KCNH2 PTGS2	31.79	0.72	gancao
MOL004806	euchrenone	KCNH2 PTGS2	30.29	0.57	gancao
MOL004808	glyasperin B	PTGS2	65.22	0.44	gancao
MOL004810	glyasperin F	PTGS2	75.84	0.54	gancao
MOL004811	Glyasperin C	KCNH2 PTGS2	45.56	0.4	gancao
MOL004814	Isotrifoliol	PTGS2	31.94	0.42	gancao
MOL004815	(E)-1-(2,4-dihydroxyphenyl)-3-(2,2-dimethylchromen-6-yl)prop-2-en-1-one	PTGS2	39.62	0.35	gancao
MOL004820	kanzonols W	PTGS2	50.48	0.52	gancao
MOL004824	(2S)-6-(2,4-dihydroxyphenyl)-2-(2-hydroxypropan-2-yl)-4-methoxy-2,3-dihydrofuro[3,2-g]chromen-7-one	PTGS2	60.25	0.63	gancao
MOL004827	Semilicoisoflavone B	PTGS2	48.78	0.55	gancao
MOL004828	Glepidotin A	PTGS2	44.72	0.35	gancao

MOL004829	Glepidotin B	PTGS2	64.46	0.34	gancao
MOL004833	Phaseolinisoflavan	PTGS2	32.01	0.45	gancao
MOL004835	Glypallichalcone	PTGS2	61.6	0.19	gancao
MOL004838	8-(6-hydroxy-2-benzofuranyl)-2,2-dimethyl-5-chromenol	PTGS2	58.44	0.38	gancao
MOL004841	Licochalcone B	PTGS2	76.76	0.19	gancao
MOL004848	licochalcone G	PTGS2	49.25	0.32	gancao
MOL004849	3-(2,4-dihydroxyphenyl)-8-(1,1-dimethylprop-2-enyl)-7-hydroxy-5-methoxy-coumarin	KCNH2<br>PTGS2	59.62	0.43	gancao
MOL004855	Licoricone	KCNH2<br>PTGS2	63.58	0.47	gancao
MOL004856	Gancaonin A	PTGS2	51.08	0.4	gancao
MOL004857	Gancaonin B	PTGS2	48.79	0.45	gancao
MOL004863	3-(3,4-dihydroxyphenyl)-5,7-dihydroxy-8-(3-methylbut-2-enyl)chromone	PTGS2	66.37	0.41	gancao
MOL004864	5,7-dihydroxy-3-(4-methoxyphenyl)-8-(3-methylbut-2-enyl)chromone	KCNH2<br>PTGS2	30.49	0.41	gancao
MOL004866	2-(3,4-dihydroxyphenyl)-5,7-dihydroxy-6-(3-methylbut-2-enyl)chromone	PTGS2	44.15	0.41	gancao
MOL004879	Glycyrin	KCNH2<br>PTGS2	52.61	0.47	gancao
MOL004883	Licoisoflavone	PTGS2	41.61	0.42	gancao
MOL004884	Licoisoflavone B	PTGS2	38.93	0.55	gancao
MOL004885	licoisoflavanone	PTGS2	52.47	0.54	gancao
MOL004891	shinpterocarpin	KCNH2<br>PTGS2	80.3	0.73	gancao
MOL004898	(E)-3-[3,4-dihydroxy-5-(3-methylbut-2-enyl)phenyl]-1-(2,4-dihydroxyphenyl)prop-2-en-1-one	PTGS2	46.27	0.31	gancao
MOL004903	liquiritin	PTGS2	65.69	0.74	gancao
MOL004904	licopyranocoumarin	PTGS2	80.36	0.65	gancao
MOL004907	Glyzaglabrin	PTGS2	61.07	0.35	gancao
MOL004908	Glabridin	PTGS2	53.25	0.47	gancao
MOL004910	Glabranin	PTGS2	52.9	0.31	gancao
MOL004911	Glabrene	PTGS2	46.27	0.44	gancao
MOL004912	Glabrone	PTGS2	52.51	0.5	gancao
MOL004915	Eurycarpin A	PTGS2	43.28	0.37	gancao
MOL004924	(-)-Medicocarpin	PTGS2	40.99	0.95	gancao
MOL004935	Sigmoidin-B	PTGS2	34.88	0.41	gancao

MOL004941	(2R)-7-hydroxy-2-(4-hydroxyphenyl)chroman-4-one	PTGS2	71.12	0.18	gancao
MOL004945	(2S)-7-hydroxy-2-(4-hydroxyphenyl)-8-(3-methylbut-2-enyl)chroman-4-one	PTGS2	36.57	0.32	gancao
MOL004948	Isoglycyrol	PTGS2	44.7	0.84	gancao
MOL004949	Isolicoflavonol	PTGS2	45.17	0.42	gancao
MOL004957	HMO	PTGS2	38.37	0.21	gancao
MOL004959	1-Methoxyphaseollidin	KCNH2<br>PTGS2	69.98	0.64	gancao
MOL004961	Quercetin der.	PTGS2	46.45	0.33	gancao
MOL004966	3'-Hydroxy-4'-O-Methylglabridin	KCNH2<br>PTGS2	43.71	0.57	gancao
MOL000497	licochalcone a	PTGS2	40.79	0.29	gancao
MOL004974	3'-Methoxyglabridin	KCNH2<br>PTGS2	46.16	0.57	gancao
MOL004978	2-[(3R)-8,8-dimethyl-3,4-dihydro-2H-pyrano[6,5-f]chromen-3-yl]-5-methoxyphenol	KCNH2<br>PTGS2	36.21	0.52	gancao
MOL004980	Inflacoumarin A	PTGS2	39.71	0.33	gancao
MOL004988	Kanzonol F	PTGS2	32.47	0.89	gancao
MOL004989	6-prenylated eriodictyol	PTGS2	39.22	0.41	gancao
MOL004990	7,2',4'-trihydroxy－5-methoxy-3－arylcoumarin	PTGS2	83.71	0.27	gancao
MOL004991	7-Acetoxy-2-methylisoflavone	PTGS2	38.92	0.26	gancao
MOL004993	8-prenylated eriodictyol	PTGS2	53.79	0.4	gancao
MOL000500	Vestitol	PTGS2	74.66	0.21	gancao
MOL005001	Gancaonin H	PTGS2	50.1	0.78	gancao
MOL005003	Licoagrocarpin	KCNH2<br>PTGS2	58.81	0.58	gancao
MOL005007	Glyasperins M	KCNH2<br>PTGS2	72.67	0.59	gancao
MOL005008	Glycyrrhiza flavonol A	PTGS2	41.28	0.6	gancao
MOL005012	Licoagroisoflavone	PTGS2	57.28	0.49	gancao
MOL005016	Odoratin	PTGS2	49.95	0.3	gancao
MOL005017	Phaseol	PTGS2	78.77	0.58	gancao
MOL005018	Xambioona	PTGS2	54.85	0.87	gancao
MOL005020	dehydroglyasperins C	PTGS2	53.82	0.37	gancao
MOL000098	quercetin	PLAT<br>PLAU<br>IGFBP3<br>FOS<br>ICAM1<br>IL1B<br>IL1A<br>EGF<br>NOS3	46.43	0.28	gancao

		MYC<br>PTGS2<br>VEGFA<br>IL6<br>CCL2<br>PRKCB<br>NFKBIA<br>COL3A1<br>CAV1<br>KCNH2<br>CYP3A4<br>HSF1<br>ABCG2<br>HAS2<br>CXCL2			
MOL002959	3'-Methoxydaidzein	PTGS2	48.57	0.24	gegen
MOL012052	Tuberosine A	PTGS2	102.67	0.34	shengma
MOL012053	cimicifugic acid	KCNH2<br>PTGS2	83.02	0.45	shengma
MOL012078	visamminol	PTGS2	50.01	0.23	shengma
MOL000449	Stigmasterol	PTGS2<br>PLAU<br>ADRA2A	43.83	0.76	shengma
MOL000483	(Z)-3-(4-hydroxy-3-methoxy-phenyl)-N-[2-(4-hydroxyphenyl)ethyl]acrylamide	PTGS2	118.35	0.26	shengma
MOL005000	Gancaonin G	PTGS2	60.44	0.39	gancao
MOL012297	puerarin	PTGS2<br>VEGFA<br>FOS<br>NFKBIA<br>NOS3<br>PLAT	24.03	0.69	gegen
MOL000390	daidzein	IL6<br>PTGS2<br>CYP3A4<br>ICAM1<br>LDLR<br>VEGFA<br>FOS<br>CAV1<br>NOS3	19.44	0.19	gegen
MOL009720	daidzin	PTGS2	14.32	0.73	gegen
MOL005928	isoferulic acid	PTGS2<br>ADRA2A	50.83	0.06	shengma

Supplementary Table S3. Top 8 hub genes

Gene name	Compounds	Degree	pathways
PTGS2	puerarin, daidzin, quercetin, daidzein, isoferulic acid, kaempferol	93	TNF signaling pathway, IL-17 signaling pathway, Kaposi sarcoma-associated herpesvirus infection, Human cytomegalovirus infection, NF-kappa B signaling pathway
IL6	paeoniflorin, daidzein, quercetin	3	TNF signaling pathway, IL-17 signaling pathway, Kaposi sarcoma-associated herpesvirus infection, Human cytomegalovirus infection
ICAM1	daidzein, quercetin, kaempferol	3	TNF signaling pathway, Kaposi sarcoma-associated herpesvirus infection, NF-kappa B signaling pathway
IL1B	quercetin	1	TNF signaling pathway, IL-17 signaling pathway, Human cytomegalovirus infection, MAPK signaling pathway,NF-kappa B signaling pathway
FOS	puerarin, daidzein, quercetin	3	IL-17 signaling pathway, Kaposi sarcoma-associated herpesvirus infection, NF-kappa B signaling pathway, MAPK signaling pathway
CCL2	quercetin	1	TNF signaling pathway, IL-17 signaling pathway, Human cytomegalovirus infection
EGF	quercetin	1	MAPK signaling pathway
VEGFA	puerarin, daidzein, quercetin	3	Kaposi sarcoma-associated herpesvirus infection, Human cytomegalovirus infection, MAPK signaling pathway

Supplementary Table S4. Details of top GO terms (BP/CC/MF)

ONTOLOGY	ID	Description	GeneRatio	BgRatio	pvalue	p.adjust	qvalue
BP	GO:0032496	response to lipopolysaccharide	6/8	330/18670	7.9223E-10	6.9213E-07	1.7359E-07
BP	GO:0002237	response to molecule of bacterial origin	6/8	343/18670	9.9946E-10	6.9213E-07	1.7359E-07
BP	GO:0010575	positive regulation of vascular endothelial growth factor production	3/8	29/18670	1.877E-07	4.2952E-05	1.0773E-05
BP	GO:0002675	positive regulation of acute inflammatory response	3/8	31/18670	2.3081E-07	4.2952E-05	1.0773E-05
BP	GO:0022407	regulation of cell-cell adhesion	5/8	403/18670	2.4269E-07	4.2952E-05	1.0773E-05
BP	GO:0045785	positive regulation of cell adhesion	5/8	403/18670	2.4269E-07	4.2952E-05	1.0773E-05
BP	GO:0051384	response to glucocorticoid	4/8	146/18670	2.4518E-07	4.2952E-05	1.0773E-05
BP	GO:0045807	positive regulation of endocytosis	4/8	153/18670	2.959E-07	4.2952E-05	1.0773E-05
BP	GO:0010574	regulation of vascular endothelial growth factor production	3/8	34/18670	3.0709E-07	4.2952E-05	1.0773E-05
BP	GO:0051090	regulation of DNA-binding transcription factor activity	5/8	432/18670	3.4274E-07	4.2952E-05	1.0773E-05

BP	GO:0010573	vascular endothelial growth factor production	3/8	36/18670	3.6626E-07	4.2952E-05	1.0773E-05
BP	GO:0031960	response to corticosteroid	4/8	162/18670	3.7215E-07	4.2952E-05	1.0773E-05
BP	GO:0061028	establishment of endothelial barrier	3/8	41/18670	5.4628E-07	5.1251E-05	1.2854E-05
BP	GO:0150077	regulation of neuroinflammatory response	3/8	41/18670	5.4628E-07	5.1251E-05	1.2854E-05
BP	GO:0071347	cellular response to interleukin-1	4/8	179/18670	5.5507E-07	5.1251E-05	1.2854E-05
BP	GO:0045429	positive regulation of nitric oxide biosynthetic process	3/8	43/18670	6.3217E-07	5.3562E-05	1.3434E-05
BP	GO:1904407	positive regulation of nitric oxide metabolic process	3/8	44/18670	6.7829E-07	5.3562E-05	1.3434E-05
BP	GO:0050900	leukocyte migration	5/8	499/18670	7.0054E-07	5.3562E-05	1.3434E-05
BP	GO:0050731	positive regulation of peptidyl-tyrosine phosphorylation	4/8	192/18670	7.3479E-07	5.3562E-05	1.3434E-05
BP	GO:0002685	regulation of leukocyte migration	4/8	196/18670	7.9793E-07	5.4737E-05	1.3728E-05
CC	GO:0031093	platelet alpha granule lumen	2/8	67/19717	0.00031433	0.0089798	0.00518359
CC	GO:0031091	platelet alpha granule	2/8	91/19717	0.00057934	0.0089798	0.00518359

CC	GO:000578 8	endoplasmic reticulum lumen	2/8	309/1971 7	0.00644 024	0.02654 45	0.01532 28
CC	GO:004512 1	membrane raft	2/8	315/1971 7	0.00668 502	0.02654 45	0.01532 28
CC	GO:009885 7	membrane microdomain	2/8	316/1971 7	0.00672 623	0.02654 45	0.01532 28
CC	GO:003477 4	secretory granule lumen	2/8	321/1971 7	0.00693 404	0.02654 45	0.01532 28
CC	GO:009858 9	membrane region	2/8	328/1971 7	0.00722 993	0.02654 45	0.01532 28
CC	GO:006020 5	cytoplasmic vesicle lumen	2/8	338/1971 7	0.00766 256	0.02654 45	0.01532 28
CC	GO:003198 3	vesicle lumen	2/8	339/1971 7	0.00770 647	0.02654 45	0.01532 28
CC	GO:000177 2	immunological synapse	1/8	36/19717	0.01451 624	0.04500 035	0.02597 643
MF	GO:007085 1	growth factor receptor binding	4/8	134/1769 7	2.1489E -07	1.1389E -05	3.393E- 06
MF	GO:004801 8	receptor ligand activity	5/8	482/1769 7	7.6832E -07	2.0361E -05	6.0657E -06
MF	GO:000512 5	cytokine activity	4/8	220/1769 7	1.5645E -06	2.764E- 05	8.2345E -06
MF	GO:000512 6	cytokine receptor binding	4/8	286/1769 7	4.4431E -06	5.8871E -05	1.7539E -05
MF	GO:000808 3	growth factor activity	3/8	163/1769 7	4.1527E -05	0.00044 018	0.00013 114
MF	GO:000517 8	integrin binding	2/8	132/1769 7	0.00150 125	0.01326 104	0.00395 066
MF	GO:003054 6	receptor activator activity	1/8	11/17697	0.00496 277	0.03691 036	0.01099 613
MF	GO:000517 2	vascular endothelial growth factor	1/8	13/17697	0.00586 277	0.03691 036	0.01099 613

		receptor binding					
MF	GO:0005161	platelet-derived growth factor receptor binding	1/8	15/17697	0.00676206	0.03691036	0.01099613
MF	GO:0005149	interleukin-1 receptor binding	1/8	16/17697	0.00721144	0.03691036	0.01099613
MF	GO:0042813	Wnt-activated receptor activity	1/8	17/17697	0.00766064	0.03691036	0.01099613
MF	GO:0030296	protein tyrosine kinase activator activity	1/8	19/17697	0.00855851	0.03780007	0.01126119
MF	GO:0070412	R-SMAD binding	1/8	23/17697	0.01035211	0.04170476	0.01242446
MF	GO:0001968	fibronectin binding	1/8	27/17697	0.01214287	0.04170476	0.01242446
MF	GO:0016702	oxidoreductase activity, acting on single donors with incorporation of molecular oxygen, incorporation of two atoms of oxygen	1/8	27/17697	0.01214287	0.04170476	0.01242446
MF	GO:0016701	oxidoreductase activity, acting on single donors with incorporation of molecular oxygen	1/8	28/17697	0.01259012	0.04170476	0.01242446
MF	GO:0005154	epidermal growth factor	1/8	33/17697	0.0148237	0.04496132	0.01339463

		receptor binding					
MF	GO:0000979	RNA polymerase II core promoter sequence-specific DNA binding	1/8	34/17697	0.01526988	0.04496132	0.01339463
MF	GO:0017147	Wnt-protein binding	1/8	37/17697	0.01660738	0.04519006	0.01346278
MF	GO:0042056	chemoattractant activity	1/8	38/17697	0.01705285	0.04519006	0.01346278
